# A Sound Body First

**Published:** 1891-01

**Authors:** Daniel Clark


### A Sound Body First.

DANIEL CLARK, M.D.

The full-limbed and chubby-faced baby who squalls and kicks with vigor, and eats enormously as it performs gymnastics on its mother's lap, is the picture of physical health; but its feeble and semi-fluid brain grows slowly, as it is needed but little at this stage of automatic life. The brain gets behind in the race of life until the muscular system develops somewhat, and thinking is needed for self-preservation. This conservation of brain force is a wise provision when taken in conjunction with comparative growth and decay. It enables us to possess vigorous brains and strong minds long after our knees are becoming weak, our hands showing signs of shakiness, our shoulders having a stoop in them and we begin to gravitate bodily toward the earth from which we sprang. As age creeps on, waste is getting the better of repair. In work there is not only a holding of the fort, but also an extension of its defences, hence the greater demand for building-up material. The boy has to grow. Mental over-strain in youth and manhood is becoming a peril to the more civilized races. This malign influence of undue mind friction, and which begins in our schools, will have its full friction in national deterioration and decay. Vice, lust, and moral corruption are largely found among the mentally defective classes. The nervous, over-strung, over-tense brain in one generation means low mentality, or ill-balanced minds in the next. This is nature's inexorable law. The only hope there is lies in the fact that the weakest go to the wall.—*The Phrenological Journal.*

